# Ethical and social difficulties of addressing environmental harms related to health research

**DOI:** 10.2471/BLT.25.294139

**Published:** 2026-03-04

**Authors:** Gabrielle Samuel, Federica Lucivero

**Affiliations:** a Department of Global Health and Social Medicine, King's College London, 30 Aldwych, London WC2B 4BG, England.; b The Oxford Centre for Ethics and Humanities and Oxford Population Health, University of Oxford, Oxford, England.

## Abstract

Health research is vital to advance human well-being, but it is also a contributor to climate change and other environmental degradation. A growing bottom-up advocacy movement is engaged in developing measures (often called tools) to help researchers better understand the ways in which they can mitigate the environmental harms associated with research. While some evidence suggests benefits of using these tools, ethical and social challenges remain. These challenges include questions about: whether these tools will place undue burdens on researchers; whether the tools will be effective in supporting large-scale mitigation of environmental harm; whether using these tools to comply with mandatory requirements will divert attention away from wider discussions about what it means to conduct research in an environmentally sustainable way; and whether these tools, which have been developed in high-income countries, reinforce existing power imbalances between high- and low-income settings and/or fail to address the needs of more marginalized research communities. In this paper, we identify and describe these ethical and social issues surrounding the use of these tools. Our aim is not to discourage their use but to urge policy-makers to reflect on these challenges as they become clearer so that tools are implemented in a way that is both effective and just.

## Résumé

La recherche en santé est essentielle pour améliorer le bien-être humain, mais elle contribue également au changement climatique et à d’autres formes de dégradation de l’environnement. Un mouvement participatif croissant de défense des intérêts s’efforce de mettre au point des mesures (souvent appelées «outils») pour aider les chercheurs à mieux comprendre comment atténuer les dommages environnementaux liés à ce type de recherche. Si certaines données suggèrent que ces outils présentent des avantages, des défis éthiques et sociaux subsistent. Ceux-ci comprennent plusieurs questions: ces outils imposeront-ils une charge de travail excessive aux chercheurs? Seront-ils efficaces pour soutenir l’atténuation à grande échelle des dommages environnementaux? Leur utilisation pour se conformer à des exigences détournera-t-elle l’attention d’un débat plus large sur les tenants et aboutissants d’une recherche en santé durable sur le plan environnemental? Dans la mesure où ils ont été élaborés dans des pays à revenu élevé, renforcent-ils les déséquilibres de pouvoir entre les contextes à revenu élevé et faible et échouent-ils à satisfaire les besoins des milieux de recherche plus marginalisés? Le présent article identifie et décrit les questions éthiques et sociales liées à l’utilisation de ces outils. Il ne vise pas à décourager leur utilisation, mais à inciter les décideurs politiques à réfléchir à ces défis à mesure qu’ils se précisent, afin de mettre en œuvre ces outils de manière à la fois efficace et équitable.

## Resumen

La investigación en salud es fundamental para promover el bienestar humano, pero también contribuye al cambio climático y a otras formas de degradación ambiental. Un creciente movimiento de incidencia desde la base participa en el desarrollo de medidas (a menudo denominadas herramientas) para ayudar a los investigadores a comprender mejor cómo pueden mitigar los daños ambientales asociados con la investigación. Aunque existen indicios de que el uso de estas herramientas puede aportar beneficios, persisten desafíos éticos y sociales. Estos desafíos incluyen interrogantes acerca de si estas herramientas impondrán cargas indebidas a los investigadores; si serán eficaces para apoyar la mitigación a gran escala de los daños ambientales; si su utilización para cumplir requisitos obligatorios desviará la atención de debates más amplios sobre lo que significa realizar investigación de manera ambientalmente sostenible; y si estas herramientas, desarrolladas en países de ingresos altos, refuerzan los desequilibrios de poder existentes entre entornos de ingresos altos y bajos o no responden a las necesidades de comunidades de investigación más marginadas. En este artículo, se identifican y describen estas cuestiones éticas y sociales relacionadas con el uso de estas herramientas. El objetivo no es desalentar su uso, sino instar a los responsables de la formulación de políticas a reflexionar sobre estos desafíos a medida que se clarifican, a fin de que las herramientas se implementen de manera eficaz y justa.

## ملخص

تعد البحوث الصحية أمرًا حيويًا لتعزيز رفاهية الإنسان، ولكنها تساهم أيضًا في تغير المناخ وتدهور البيئة. هناك حركة تأييد متنامية من القاعدة إلى القمة تعمل على تطوير تدابير (غالبًا ما تسمى أدوات) لمساعدة الباحثين على تحقيق فهم أفضل للطرق التي يمكنهم من خلالها التخفيف من الأضرار البيئية المرتبطة بالأبحاث. وبينما تشير بعض الأدلة إلى فوائد استخدام هذه الأدوات، إلا أنه تظل هناك تحديات أخلاقية واجتماعية. وتشمل هذه التحديات أسئلة تدور حول: ما إذا كانت هذه الأدوات ستفرض أعباءً غير مبررة على الباحثين؛ وما إذا كانت الأدوات ستكون فعالة في دعم التخفيف على نطاق واسع من الأضرار البيئية؛ وما إذا كان استخدام هذه الأدوات للامتثال للمتطلبات الإلزامية سيصرف الانتباه عن المناقشات الأوسع نطاقًا حول ما يعنيه إجراء البحوث بطريقة مستدامة بيئيًا؛ وهل هذه الأدوات، التي تم تطويرها في الدول ذات الدخل المرتفع، تعزز اختلالات التوازن الحالية في القوة بين البيئات ذات الدخل المرتفع والبيئات ذات الدخل المنخفض و/أو تفشل في تلبية احتياجات مجتمعات البحث الأكثر تهميشًا. في هذه الورقة البحثية، نحن نحدد ونصف هذه القضايا الأخلاقية والاجتماعية المحيطة باستخدام هذه الأدوات. نحن لا نهدف إلى تثبيط استخدامها، ولكننا نحث واضعي السياسات على التفكير في هذه التحديات مع تزايد وضوحها، بحيث يتم تنفيذ الأدوات بطريقة فعالة وعادلة.

## 摘要

健康研究对于提升人类福祉而言至关重要，但同时它也是导致气候变化及其他环境退化现象的因素之一。一场正处于蓬勃发展阶段的自下而上的倡导运动旨在开发相应衡量标准（通常被称为工具），以帮助研究人员进一步了解其可采取哪些方式来减轻研究相关环境危害。尽管有证据表明使用这些工具能带来各种好处，但伦理和社会挑战始终存在。这些挑战包括以下相关问题：这些工具是否会对研究人员造成过度负担；这些工具能否有效地支持大幅减轻环境危害；使用这些工具以遵循相关强制规定，是否会转移在更广泛地讨论以环境上可持续的方式开展研究所代表的含义方面的注意力；以及在高收入国家开发的这些工具是否会导致高收入地区与低收入地区之间的现有权力失衡状况加剧和/或是否无法满足更多边缘化研究群体的需求。在本文中，我们指出并详细说明了因使用这些工具而导致的此类伦理和社会问题。我们的目标并非阻止使用这些工具，而是敦促决策者在这**些挑战变得更加明朗时能予以仔细考虑，从而确保以公正有效的方式使用这些工具。**

## Резюме

Исследования в области здравоохранения жизненно важны для улучшения благополучия людей, однако они также способствуют изменению климата и иным формам деградации окружающей среды. Формируется растущее низовое движение в защиту экологии, которое разрабатывает меры (часто называемые инструментами), призванные помочь исследователям лучше понять, каким образом можно сократить связанный с исследованиями экологический вред. Хотя некоторые данные свидетельствуют о преимуществах использования этих инструментов, сохраняются этические и социальные проблемы. В числе этих проблем поднимаются такие вопросы: будут ли эти инструменты создавать чрезмерную нагрузку на исследователей; окажутся ли они эффективными для поддержки масштабного сокращения экологического вреда; не приведет ли их использование с целью соблюдения обязательных требований к отвлечению внимания от более широких дискуссий о том, что означает проводить исследования с соблюдением принципов экологической устойчивости; не усиливают ли эти инструменты, разработанные в странах с высоким уровнем дохода, существующий дисбаланс сил между условиями с высоким и низким уровнем доходов; учитываются ли в должной мере потребности более маргинализированных исследовательских сообществ. В данной статье выявлены и описаны этические и социальные вопросы, связанные с применением указанных инструментов. Наша цель не в том, чтобы отговаривать от их использования, а в том, чтобы побудить лиц, принимающих решения, учитывать эти проблемы по мере их прояснения, чтобы внедрение инструментов было эффективным и справедливым.

## Introduction

Health research contributes to climate change and other environmental harms. Given the relationship between environmental harms and human ill-health,^1^ bioethicists are increasingly interested in moral questions about if and how the health research community should tackle these harms. Particular attention has focused on whether a moral obligation to mitigate such harms exists and, if so, what this obligation should look like in practice.^2^^–^^4^ Despite some disagreement,^5^ growing consensus supports such an obligation, provided it does not compromise research integrity. This argument has been framed as a matter of international priority, as a commitment to human health (including planetary health) and/or as a commitment to health equity and justice.^2^^,^^6^^,^^7^

Alongside, a growing bottom-up advocacy movement is engaged in developing measures (often called tools) to help researchers better understand the ways in which their practices contribute to adverse environmental impacts, with the aim of encouraging researchers to mitigate these harms. Many tools have been developed which can be divided into two main categories (Table 1). First, tools that allow researchers to assess the carbon dioxide equivalent emissions (hereafter referred to as emissions) associated with their research practices. For example, carbon calculators have been developed for researchers training machine learning algorithms so that they can predict the likely emissions associated with algorithmic training, and then (it is hoped) optimize algorithmic design to reduce energy consumption.^15^ In another example, carbon accounting methods for clinical trials have been developed to help triallists predict emission hot spots. Hot spots often relate to travel, with online appointments and meetings offering an option for emission reductions.^16^

**Table 1:** Examples of tools developed to help researchers reduce the environmental harms associated with their practices

Research setting	Environmental harms	Tools	
		Carbon assessment tool	Accreditation system
Wet laboratory (where experiments using reagents and samples take place)	• Use and disposal of chemicals and biological materials • Energy (including for ultra-low temperature sample storage) • Water use • Use of single-use plastics	GES 1point5 (France).^8^ The tool calculates carbon emissions associated with laboratories	• LEAF: Laboratory Efficiency Assessment Framework (United Kingdom).^9^ The framework has 48 measures to implement to reduce environmental harms and has three certification levels: bronze (16 measures), silver (an additional 17 measures), and gold (a further 15 measures). Certification is valid for 1 year. • My Green Lab® (United States).^10^ The organization conducts baseline surveys about laboratory practices with feedback provided. Laboratories repeat assessment and depending on their new score, achieve a certain standard. Certification is valid for 2 years. • EFLM: Green & Sustainable Laboratories (Europe).^11^ The certification focuses on energy, water, waste and chemical management. Applications that achieve at least 75% for each section are certified as Green & Sustainable Laboratories
Dry laboratory (spaces dedicated to computational research)	• Energy use to generate, store and process data • Reliance on mining for manufacture of digital hardware and infrastructures • Electronic waste production	Green Algorithms.^12^ The project offers calculators to assess emissions associated with training algorithms (United Kingdom)	• Green DISC (United Kingdom).^13^ The scheme was developed using a similar model to LEAF
Clinical trials	• Energy consumption • Hazardous and nonhazardous waste, including pharmaceuticals • Air pollution due to travel • Water use	Low Carbon Clinical Trials Group.^14^ The group provides a method for quantifying the carbon footprint of clinical trials (United Kingdom)	NA

NA: not applicable.

Second, accreditation systems have been developed that require researchers to adhere to practices that mitigate research-associated environmental harms. Examples of practices include: recycling; reducing energy consumption by turning lights and equipment off when rooms are empty; lowering fume hoods when not in use to save energy; reducing the use of single-use plastics; and awareness-building for new laboratory researchers. Once assessment is complete and mitigating practices and processes are in place, laboratories receive an award or certification. Globally, the most well-known accreditation systems include My Green Lab® and the Laboratory Efficiency Assessment Framework (LEAF; Table 1).^9^^,^^10^ Some evidence suggests that such accreditation programmes are effective, with small-scale studies showing increased motivation to tackle environmental harms, increases in recycling and decreases in laboratory-associated energy consumption.^17^^,^^18^ In 2021, 20 AstraZeneca research and development laboratories reported a saving of an estimated 1 270 185 kWh/year (4 572 666 MJ/year) after implementing the My Green Lab® accreditation standard, equivalent to 900 tonnes of emissions.^19^ In LEAF’s pilot, across 2 years, organizations were reported to have saved about 2.9 tonnes of emissions.^19^

Globally, the use of such tools remains voluntary (except for researchers applying to some funding bodies in the United Kingdom of Great Britain and Northern Ireland), although uptake is increasing. While few instances exist of the use of carbon calculators for greener trials,^16^ LEAF is used in more than 15 countries and 95 institutions^9^ and My Green Lab® is used in more than 4500 laboratories worldwide, with 9500 ambassadors trained in more than 90 countries. My Green Lab® has a particularly strong presence in the private sector. As the frequency of tool use, as well as the number of tools available, continues to grow, there has been a push to increase the use of these tools through regulation.^20^^,^^21^ As such, it is vital that ethical and social issues associated with the use of these tools are examined, particularly on questions of tool effectiveness and justice. In terms of justice, this imperative is important because these tools have so far only been developed in high-income countries, particularly the United Kingdom and the United States of America, and the European Region. In this article, we describe these ethical and social issues, emphasizing the need for the careful use of these tools if we are to ensure their continued development and proliferation in an effective and just way. In the following sections, we describe three main considerations related to: (i) responsibilities; (ii) tool effectiveness (including issues associated with behaviour change, rebound effects, research quality and compliance-based approaches); and (iii) social and environmental justice concerns given that these tools have been developed in high-income settings. Our aim is not to discourage the use of such tools, but to identify, as much as possible, the ethical and social issues that must be considered as these tools are increasingly adopted in health research. By mapping these concerns, our aim is to raise awareness of them among implementers and enable reflection when using the tools. As the field evolves, it will become clearer how and where the issues we raise are seen and how to address them.

## Responsibility

Tools have been developed on the premise that researchers have a responsibility to mitigate the potential environmental harms of their research practices. A case has been made for this premise by arguing that everyone has a duty to reduce their negative environmental impacts as we should all take collective responsibility for climate change and environmental degradation.^22^ However, this argument may place undue burdens on researchers and unintentionally shift responsibility away from the broader structural, institutional and geopolitical factors associated with the environmental harms of research.^23^ These factors include, for example, institutional regulation, legislation and broader changes to pro-innovation, neo-liberal models of governance that prioritize consumption. Addressing these factors would likely be more effective in mitigating harms than efforts at the individual research level. These discussions are well established in the literature.^2^^,^^24^^–^^26^

## Effectiveness

Effectiveness is a key aspect of tool use: ineffective tools lead to wasted time and resources. In the following subsections, we describe various factors that need to be considered by tool implementers (individuals, institutions, funding bodies and policy-makers) if tools are to be as effective as possible. These factors include the need to ensure that: (i) the use of a tool leads to relevant behaviour change; (ii) behaviour change based on the use of a tool does not unintentionally increase environmental harms in other areas; (iii) the use of a tool does not distract from the fact that a key way to undertake environmentally sustainable research is to conduct research that has social value, is rigorous and is of good quality; and (iv) the use of tools does not mask the need to reflect on environmental harms that cannot be mitigated by using the tools.

### Behaviour change

The development of tools is driven by an expectation that researchers will change their behaviour following the use of a tool, and this change will lead to real reductions in research-related environmental harms (that is, tools will be effective). However, individual, social and political factors complicate this assumption. A wide range of behaviour change models have long complicated simplistic presumptions that behaviour change is straightforward. These models point to, for example, the role of collective action, issues with institutional change and inertia, and the importance of the political economy, behavioural nudges, social norms and economic incentives.^27^^–^^29^ The widely accepted Capabilities, Opportunities, Motivations, Behaviour (COM-B) model,^30^ while criticized,^31^ draws attention to these limitations. COM-B attempts to incorporate the wide range of factors affecting behaviour change into one framework, claiming that in any setting, behaviour change requires individuals to have the capability (ability), opportunity and motivation (willingness) to change. Capability refers to whether researchers know how to address environmental harms associated with research. The use of tools might be one example of this capability, as would training courses to teach researchers how to use tools, meetings and online forums to share ideas, or through spontaneous thinking (for example, choosing to recycle). Opportunity refers to the social, political, cultural and organizational factors within health research settings that allow researchers to make changes, including the support of research institutions for the reduction of the adverse environmental harms of research through policies and practices. In some countries, such as the United Kingdom, such policies are now being enforced. 

Finally, even with the ability and opportunity, changes in researcher behaviour will not happen without motivation. Research in high-income countries has shown that some health researchers think environmental harms fall outside of their responsibility,^5^ or that, even when they want to address environmental harms, mitigating these harms requires too much time and effort as other motivations are more important. These other motivations include the need to conduct and publish research at a fast pace, the need to secure more funding and other work-based demands. Together, these factors make it difficult for researchers to prioritize behaviour change.^32^^,^^33^ Furthermore, interviews with health researchers working in dry laboratories in Kenya and Senegal suggest little motivation to tackle the environmental harms of research because of the need to prioritize the many immediate unmet health needs in these countries, and because researchers do not consider that their countries are responsible for these harms.^34^ As such, while tools are being developed to reduce the environmental harms of research, behavioural change is a substantial barrier that will need to be overcome on a large scale. Furthermore, questions remain about which researchers, in which country or region, should have to change their behaviour given long-standing climate and environmental justice issues across different geographic contexts.

### Rebound effects

Even if researchers change their behaviour in response to the use of tools, this change may not be sufficient to achieve sector-wide reductions in environmental harms. This outcome is because single tools will only be able to address some specific environmental harms. For example, if behaviour changes in one area inadvertently alter behaviour elsewhere, this change may lead to increases in consumption or at least to lower-than expected reductions. This scenario is now a well-established phenomenon known as the rebound effect,^24^^,^^35^ and illustrates the need to make clear the interconnections between reductions in environmental harms from tool use and any resultant behaviour changes in response to these reductions. To provide an everyday example, a state may choose to improve the efficiency of its currently congested roads by building more roads. However, without constraining how many vehicles can be bought by consumers, more roads may lead to the purchase of more vehicles and ultimately more traffic, which leads to more congestion. An approach that recognizes these interdependencies and adjusts for them is likely to be more effective in achieving the desired outcome.

### Rigorous research

Another implicit assumption is that the tools are the most appropriate way to fix environmentally harmful research practices. This assumption may distract from the importance of robust research practices, such as appropriate research design, planning and reproducibility, as a key component of reducing environmental harm. Less rigorous research practices can lead to unnecessary use and waste of resources.^36^ Furthermore, any lack of reproducibility, or lack of correction for irreproducibility, means that any efforts to reduce environmental harms associated with the research process are wasted because the research process itself has no value.^37^ As such, consideration of the environmental harms of health research should not be a separate addition to the research process through tool use, but should be implicit in the research design. Rigorous, well-planned research based on robust methods will produce less environmental harm, or at least more proportional benefit, because it reduces unnecessary research practices, thereby reducing research waste and environmental waste, while delivering reproducible research and reliable results.

### Compliance-based approaches

As more tools are developed in high-income countries, some high-resource settings are shifting towards the mandatory use of tools through top-down policies. In the United Kingdom, two large health research funding bodies (Wellcome and Cancer Research United Kingdom) now require fund recipients to work in research laboratories with recognized environmental sustainability accreditation from, for example, LEAF and My Green Lab®. In addition, some research councils in the United Kingdom have piloted or now include environmental assessment criteria for funding proposals. Furthermore, funding bodies alongside other United Kingdom research institutions have developed an agreement for environmentally sustainable research that is targeted at other funding bodies, higher education institutions and researchers.^20^ In the European Region, other countries are exploring or have implemented measures to encourage environmentally sustainable research. For example, Science Foundation Ireland is piloting My Green Lab®; the European Commission’s Marie Skłodowska-Curie Actions Fellowship has a code of good practice for environmentally sustainable research (Green Charter); the German Science Foundation and Foundation for Polish Science have an environmental assessment criterion within funding application forms; and the French government requires artificial intelligence-related applications in national funding calls to include carbon footprint estimates of their models.^38^ Outside Europe, environmental considerations in other countries include the Democratic Republic of the Congo’s *Guidelines for the ethical review of research involving human subjects in DR Congo*, which requires a “preliminary assessment of economic, social, cultural and likely environmental factors” in the context of collective consent when conducting research involving “traditional knowledge or the sacred rights of an indigenous community.”^39^

Together, these compliance-based policies are important steps towards tackling environmental harms associated with health research quickly and effectively. At the same time, and while the implementation of these policies is too recent to evaluate, we must not fall into the assumption that policies alone can address complex environmental issues. Compliance-based approaches can hinder the reflective capabilities of individuals, communities and organizations to engage with broader value-centred questions.^23^ These questions include: what environmental sustainability means to the health research community(ies); what the health research community(ies) wants to sustain and how; and how the community(ies) thinks tensions between different practices or values systems (economic, financial, personal and environmental) should be addressed? These reflective capabilities are particularly important if compliance-based policies are exported to other countries, such as low- and middle-income countries whose communities have different meanings and understandings of environmental sustainability. 

Furthermore, compliance-based policies might also become viewed as a bureaucratic hurdle imposed in a top-down fashion,^40^ or might lead to researchers relinquishing their responsibilities once a regulation is followed, because they consider their responsibilities are over and there is no need for on-going reflection through the research process – an issue that has been seen in research ethics regulation.^41^ Finally, in extreme cases, a rule-compliance focus can lead to adverse outcomes; for example, there are instances of researchers reporting important health research studies with real clinical benefit having been held up because of questions about compliance-based consent.^42^ Thus, implementing a compliance-based approach is important, but must not be the only mechanism for tackling the environmental harms of research.

## Tool development

To date, tools have only been developed in high-income countries and most uptake appears to have been in these countries, although mention of the environmental harms of research has been made in research policy guidance in some low- and middle-income countries.^39^ As mentioned earlier, developing tools in high-income settings and exporting them to another context, whether that be low- and middle-income countries or underserved communities in high-income countries, raises ethical and cultural concerns. This concern is explained by the fact that research in the social sciences, philosophy and ethics^43^^,^^44^ has repeatedly shown how different research communities often have distinct values and practices, even if there is a wish to address environmental harms in these countries. If this situation exists (it is open to empirical investigation), then three key ethical concerns arise. First, tools (whether a carbon calculator or assessment method) may raise different social, ethical and practical issues if their use affects the practices of local researchers and/or exacerbates existing inequalities due to undue burdens on researchers.^45^^,^^46^ For example, while decentralized trial design can co-beneficially mitigate the environmental harms associated with clinical trials, a pre-existing lack of digital infrastructure and digital literacy is a barrier to implementing decentralized trials in many low- and middle-income countries and some regions of upper middle-income countries and high-income countries. Implementing decentralized trials could exacerbate social injustice related to the digital divide, meaning that potential participants in underserved regions may have less opportunity to take part in such trials. This approach could also disadvantage clinical trial staff who are not digitally literate. In another example, the types of practice required to complete accreditation awards may not be available to researchers in resource-constrained settings, including, for example, access to supply chains for more eco-friendly materials and/or appropriate recycling and disposal processes.

Second, a tool’s assessment criteria may not align with the priorities of all countries. For instance, Chile has high renewable energy dependence, but the country is burdened by environmental pollutants associated with mining, particularly for the digital sector.^47^ For this country, the use of carbon calculators may be less relevant than tools that assess other environmental factors. This scenario speaks to a more general concern that the focus on carbon reduction in instruments tackling climate-change reduction has led to so-called climate tunnel vision^48^ and net zero dashboards^49^ that pay attention to modelled calculations of carbon rather than more holistically addressing environmental concerns. Third, some research practices outside of high-income settings may include ways of knowing that may have low environmental harms. Focusing on tools may distract us from paying attention to these ways of knowing. For example, facilities in low- and middle-income countries may already operate under the principle of resourcefulness in response to a lack of human and material resources. This situation may have resulted in the development of innovative, less costly and, accidentally, more environmentally friendly solutions. Some examples include sharing reagents to reduce leftovers, reusing equipment which otherwise might be unnecessarily disposed of, and washing hands as a substitute for plastic gloves where appropriate. Focusing on tools that have been developed in high-income countries may distract from capturing and learning from these ways of knowing. Finally, broader ways of knowing and understanding the environment may exist in different countries in which health research sites operate, including knowledge aligned with Indigenous peoples and other communities with their own cultural context. This knowledge and understanding may not align with the implicit norms of tools, and needs to be taken into consideration.

Given these issues, we must be careful not to export what it means to conduct health research in an environmentally sustainable way in high-income settings to other countries. Understanding what these tools can and cannot do, and how they may work in specific contexts and communities is important to ensure their effectiveness. The use of tools must reflect the cultural context in which they are used to ensure fair and just implementation. This placement must only be done through engagement with (at a minimum) and leadership (at a maximum) from local communities. In these situations, attention must also be paid to what researchers in high-income settings can learn from other countries about how best to reduce research consumption. This approach includes valuing low-waste innovations developed in settings with constrained infrastructure and recognizing them as legitimate, efficient models from which all researchers can learn. This approach is not about accepting lower scientific or safety standards, but realizing that the dominant high-resource methods are not necessarily more effective than evidence-based lower-resource methods. Adopting this approach should be seen as a way to benefit from valuable innovations and promote equitable, global benchmarks. However, doing so will be a challenge as it will require countering dominant consumption practices and capitalist markets in high-income settings. Overall, more research and multicountry co-learning is needed to better understand how to address these issues in practice. This research is now starting,^50^ including guidance from the World Health Organization on how best to design and conduct clinical trials in an environmentally sustainable way.

## Conclusion

Although we acknowledge the advantage of requiring the use of tools to tackle the environmental harms of research, in our analysis we reflect on the importance of critically evaluating these tools with respect to their effectiveness and fairness. We call on funders to verify that these tools are fit for purpose in different contexts and to ascertain that they do not amplify injustices in research endeavours. Both funders and research organizations must also ensure that the use of tools does not become another so-called tick-box exercise that diverts researchers away from reflecting on the need for wider systemic change in research processes. Publishers and funders must make sure that the use of tools does not distract from the fact that environmental considerations are central to and not separate from assuring rigorous research. Ensuring all these measures are considered will allow us to view tools as just one instrument in a toolbox needed to re-imagine how research is conducted, so that overall consumption and waste are reduced and benefit from research is maximized.
